# Fowlerstefin, a cysteine protease inhibitor of *Naegleria fowleri*, induces inflammatory responses in BV-2 microglial cells *in vitro*

**DOI:** 10.1186/s13071-020-3909-6

**Published:** 2020-01-29

**Authors:** Thị Lam Thái, Jung-Mi Kang, Hương Giang Lê, Jinyoung Lee, Won Gi Yoo, Ho-Joon Shin, Woon-Mok Sohn, Byoung-Kuk Na

**Affiliations:** 1 Department of Parasitology and Tropical Medicine and Institute of Health Sciences, Gyeongsang National University College of Medicine, Jinju, 52727 Republic of Korea; 2 BK21Plus Team for Anti-aging Biotechnology and Industry, Department of Convergence Medical Science, Gyeongsang National University, Jinju, 52727 Republic of Korea; 3 Department of Tropical Medicine and Inha Research Institute for Medical Sciences, Inha University School of Medicine, Incheon, 22212 Republic of Korea; 4 Department of Medical Environmental Biology, Chung-Ang University College of Medicine, Seoul, 06974 Republic of Korea; 5 Department of Microbiology, Ajou University School of Medicine, Suwon, 16499 Republic of Korea; 6 Department of Biomedical Science, Ajou University School of Medicine, Suwon, 16499 Republic of Korea

**Keywords:** *Naegleria fowleri*, Cysteine protease inhibitor, Cysteine proteases, Microglial cells, Inflammatory response

## Abstract

**Background:**

*Naegleria fowleri* is a free-living amoeba that causes an opportunistic fatal infection known as primary amoebic meningoencephalitis (PAM) in humans. Cysteine proteases produced by the amoeba may play critical roles in the pathogenesis of infection. In this study, a novel cysteine protease inhibitor of *N. fowleri* (fowlerstefin) was characterized to elucidate its biological function as an endogenous cysteine protease inhibitor of the parasite as well as a pathogenic molecule that induces immune responses in microglial cells.

**Methods:**

Recombinant fowlerstefin was expressed in *Escherichia coli*. The inhibitory activity of fowlerstefin against several cysteine proteases, including human cathepsins B and L, papain and NfCPB-L, was analyzed. Fowlerstefin-induced pro-inflammatory response in BV-2 microglial cells was anayzed by cytokine array assay, reverse transcription polymerase chain reaction, and enzyme-linked immunosorbent assay.

**Results:**

Fowlerstefin is a cysteine protease inhibitor with a monomeric structure, and belongs to the stefin family. Recombinant fowlerstefin effectively inhibited diverse cysteine proteases including cathepsin B-like cysteine proteases of *N. fowleri* (NfCPB-L), human cathepsins B and L, and papain. Expression of fowlerstefin in the amoeba was optimal during the trophozoite stage and gradually decreased in cysts. Fowlerstefin induced an inflammatory response in BV-2 microglial cells. Fowlerstefin induced the expression of several pro-inflammatory cytokines and chemokines including IL-6 and TNF in BV-2 microglial cells. Fowlerstefin-induced expression of IL-6 and TNF in BV-2 microglial cells was regulated by mitogen-activated protein kinase (MAPKs). The inflammatory response induced by fowlerstefin in BV-2 microglial cells was downregulated *via* inhibition of NF-κB and AP-1.

**Conclusions:**

Fowlerstefin is a pathogenic molecule that stimulates BV-2 microglial cells to produce pro-inflammatory cytokines through NF-κB- and AP-1-dependent MAPK signaling pathways. Fowlerstefin-induced inflammatory cytokines exacerbate the inflammatory response in *N. fowleri-*infected areas and contribute to the pathogenesis of PAM.

## Background

*Naegleria fowleri* is a free-living amoeba that causes a lethal brain infection known as primary amoebic meningoencephalitis (PAM) in humans [1–3]. The amoeba is ubiquitous and is usually found in diverse environments such as fresh water lakes, rivers, ponds, hot springs and unchlorinated or minimally-chlorinated swimming pools [1, 4, 5]. Most PAM cases have been reported in children and young individuals who recently swam in warm freshwater and the concern due to the disease has been increasing in subtropical and tropical areas [4, 6–8]. *Naegleria fowleri* infection is initiated by inhaling water containing amoebae into the host nasal cavity. The inhaled amoebae pass the respiratory epithelium and olfactory mucosa and then migrate through the cribriform plate into the brain [9]. Within the brain, the amoebae trigger extensive tissue damage along with acute inflammation. The initial symptoms of the infection include fever, headache, nausea, vomiting, stiff neck, confusion and occasional seizures [2, 10]. The acute hemorrhagic meningoencephalitis that follows invasion of the central nervous system (CNS) generally results in death within 7–10 days of infection [10].

PAM is difficult to treat due to the rapid disease progression and the lack of diagnostic tools in the early phase and effective therapeutic agents. Understanding the molecular mechanism of PAM induced by *N. fowleri* is important in order to develop effective diagnostic or therapeutic interventions targeting PAM. It has been proposed that PAM may be induced by both contact-dependent and contact-independent mechanisms by *N. fowleri*, which eventually results in host cell death and inflammatory response [9]. In the contact-dependent mechanism, *N. fowleri* trophozoites directly destroy the target host cells *via* trogocytosis, involving food-cup formation on the amoeba surface and the release of cytolytic molecules [9]. Several proteins including Nfa1, Nf-actin and heat-shock protein 70 may play essential roles in the phagocytic food-cup formation and in adaptive survival of the amoeba [11–13]. In the contact-independent mechanism, the excretory and secretory proteins (ESP) of *N. fowleri* are likely to play a critical role in inducing cytopathic effect against the target host cells or inflammatory response [14–18].

Proteases are ubiquitous enzymes that play pivotal roles in the pathogenesis and physiology of parasitic organisms [19–22]. Thus, these enzymes are promising targets for vaccine or drug development. Recently, two novel cathepsin B-like cysteine proteases of *N. fowleri* (NfCPBs), known as NfCPB and NfCPB-L, have been identified and their biochemical properties were partially characterized [23]. The two NfCPBs are actively secreted or released from *N. fowleri* trophozoites and play a critical role in host tissue invasion and immune evasion by the amoeba. Although the enzymes play important roles in *N. fowleri* biology and pathogenecity, a strict regulation of their activities is essential to minimize inadequate superfluous damage to the parasite. However, the mechanisms used by the amoeba to control protease activity have not been understood. In this study, a novel cysteine protease inhibitor of *N. fowleri*, named fowlerstefin, was identified and its biochemical and immunological properties were characterized.

## Methods

### Culture and maintenance of *Naegleria fowleri*

*Naegleria fowleri* (Carter NF69 strain, ATCC no. 30215) was cultured axenically in Nelsonʼs medium supplemented with 5% fetal bovine serum (FBS; Gibco, Rockville, Maryland, USA) and 1% penicillin/streptomycin at 37 °C [24]. The amoebae were usually sub-cultured every 3 days with the same media and used in this study.

### Cloning a gene encoding fowlerstefin

Trophozoites of *N. fowleri* were collected by centrifugation and rinsed with warm phosphate-buffered saline (PBS, pH 7.4) several times. Total RNAs were isolated by using TRIzol reagent (Invitrogen, Carlsbad, California, USA) according to the manufacturer’s protocols. The purified total RNA was treated with RNase-free DNase (Gibco) to remove any contaminating DNA. The cDNAs were synthesized from the total RNA (2 μg) using a RNA to cDNA EcoDry Premix Kit (Clontech, Mountain View, California, USA) followed by the manufacturer’s instructions. A gene encoding a cysteine protease inhibitor (gene ID: NF0067710) was found by data mining the *N. fowleri* genomic resource (AmoebaDB, http://amoebadb.org/amoeba/). Fowlerstefin gene was amplified by polymerase chain reaction (PCR) using the primers (5ʹ-ATG AAG AAA ATC ATT CTT GTT GCC TTG-3ʹ and 5ʹ-TTA TCT TCG TTC AGA AAC AGA GAC CAA A-3ʹ) and *N. fowleri* cDNA. The amplification was carried out with the thermal cycling condition: one cycle of an initial denaturation 95 °C for 5 min, 30 cycles at 95 °C for 1 min, 52 °C for 1 min and 72 °C for 1 min, followed by a final extension step at 72 °C for 10 min. The PCR product was separated by 1.5% agarose gel electrophoresis and the amplified product was purified and ligated into the T&A cloning vector (Real Biotech Corporation, Banqiao City, Taiwan). The ligation mixture was transformed into competent *Escherichia coli* DH5α cells. Positive clones harboring the appropriate insert were screened by colony PCR. The nucleotide sequence of the insert was determined by automated sequencing. The primary structure of the deduced amino acid sequence was analyzed with DNASTAR (DNASTAR, Madison, WI, USA) and Signal P (http://www.cbs.dtu.dk/services/SignalP/). The phylogenetic tree was constructed using the neighbor-joining method with the MEGA 4 program (http://www.megasoftware.net). The robustness of the nodes were assessed with 1000 bootstrap replications.

### Production and purification of recombinant fowlerstefin

To produce recombinant fowlerstefin, a parial gene of fowlerstefin lacking the region encoding signal peptide was amplified by PCR using the following primers: 5ʹ-GGA TCC AGT GTT GTT CCT GGT GGG-3ʹ containing a *Bam*HI site at 5ʹ end and 5ʹ-AAG CTT TTA TCT TCG TTC AGA AAC-3ʹ containing a *Hind*III site at 5ʹ end. The amplified product was analyzed on 1.5% agarose gel, purified from the gel, ligated into the T&A cloning vector (Real Biotech Corporation) and transformed into *E.coli* DH5α. The resulting plasmid DNA was digested with *Bam*HI and *Hind*III, cloned into the corresponding restriction enzyme sites of the pQE-9 expression vector (Qiagen, Hilden, Germany) and transformed into *E. coli* M15 [pREP4] cells (Qiagen). The *E. coli* clone was cultured in Luria Bertani broth and the expression of the recombinant protein was induced with 1 mM isopropyl-1-thio-β-D-galactopyranoside (IPTG) at 37 °C for 3 h. The cultured cells were harvested, suspended in native lysis buffer (50 mM NaH_2_PO_4_, 300 mM NaCl, 10 mM imidazole, pH 8.0), sonicated on ice and then centrifuged at 4 °C for 30 min at 12,000×*g*. The recombinant protein was purified by nickel-nitrilotriacetic acid (Ni-NTA) chromatography (Qiagen) according to the protocols provided by the manufacturer. In brief, the recombinant fowlerstefin was eluted with two different elution buffers, buffer 1 (50 mM NaH_2_PO_4_, 300 mM NaCl, 100 mM imidazole, pH 8.0) and buffer 2 (50 mM NaH_2_PO_4_, 300 mM NaCl, 250 mM imidazole, pH 8.0), to eliminate contamination of non-specific bound proteins. Samples taken at each elution peak were pooled and the purity of the recombinant fowlerstefin was analyzed *via* 15% sodium dodecyl sulfate-polyacrylamide gel electrophoresis (SDS-PAGE). Protein concentration was assayed with Pierce^TM^ BCA Protein Assay Kit (Pierce, Rockford, lL, USA). To remove any LPS contamination in the Ni-NTA affinity purified fowlerstefin, the Detoxi-gel endotoxin removing column (Pierce) was used followed by the manufacturer’s protocols. Residual amount of endotoxin was confirmed by Pierce™ LAL Chromogenic Endotoxin Quantitation Kit (Pierce). The residaul amount of endotoxin was less than 0.05 EU when detemined by the chromogenic assay. The LPS-depleted fowlerstefin was filtered with a sterile syringe filter (0.22 μm; Millipore, Billerica, MA, USA) and used for further experiments.

### Production of polyclonal antibody for fowlerstefin (anti-fowlerstefin)

Anti-fowlerstefin antibody was produced by immunizing two BALB/c mice intraperitoneally with the purified recombinant fowlerstefin (50 µg) for three times every 2 weeks. The purified recombinant fowlerstefin was mixed with the same volume of Freund’s complete adjuvant (Sigma-Aldrich, St. Louis, MO, USA) for the first immunization and Freund’s incomplete adjuvant (Sigma-Aldrich) for the two booster injections. Two weeks after the final immunization, the mice were sacrificed and their sera were collected. The immunoglobulin G (IgG) fraction was purified with a Protein G-Sepharose (Amersham Biosciences, Piscataway, NJ, USA) according to the manufacturer’s protocols. The specificity of the antibody was confirmed by immunoblot (Additional file 1: Figure S1).

### Inhibitory activity of fowlerstefin

The inhibitory activity of fowlerstefin against several cysteine proteases, including human cathepsin B (HCB; Sigma-Aldrich), human cathepsin L (HCL; Sigma-Aldrich), papain (Sigma-Aldrich) and NfCPB-L, was analyzed by assaying the residual enzyme activity after incubation of each enzyme with fowlerstefin. Each enzyme (20 nM) was incubated with different concentrations (0–100 nM) of fowlerstefin in 50 mM sodium phosphate (pH 6.0) for 30 min at 37 °C. The concentration of each enzyme was deretmined by active site titration with transepoxy-succinyl-l-leucylamido(4-guanidino) butane (E-64; Sigma-Aldrich) [25]. Substrate solution was added to the mixture and the residual enzyme activity was assayed by measuring the released fluorescence (excitation at 355 nm; emission at 460 nm) with a Fluoroskan Ascent FL (Thermo Fisher Scientific, Vantaa, Finland). The substrate was benzyloxycarbonyl-_L_-leucyl-_L_-arginine 4-methyl-coumaryl-7-amide (Z-LR-MCA; Peptide International, Louisville, KY, USA) and the assay buffers for each enzyme were as follows: HCB and HCL, 50 mM sodium acetate (pH 6.0); papain, 50 mM sodium acetate (pH 5.0); and NfCPB-L, 50 mM sodium acetate (pH 4.5). All assay buffers contained 10 mM dithiothreitol (DTT). Recombinant NfCPB-L was produced using the method described previously [23]. In all assays, E-64 was used as a control inhibitor. All the assays were conducted in triplicate and the mean and standard deviation (SD) were calculated.

### pH dependent inhibiton and stability of fowlerstefin

The effect of pH on the inhibitory activity of fowlerstefin was measured by incubating the purified fowlerstefin (20 nM) with the same concentration of HCB, HCL, papain or NfCPB-L in different pH buffers [50 mM sodium acetate (pH 4.0–5.0) or 50 mM sodium phosphate (pH 6.0–7.0)] at 37 °C for 30 min. After incubation, the enzyme activity of each sample was assayed as described above. The thermal stability of fowlerstefin was determined by incubation at different temperatures (37 °C and 95 °C) for 1 to 3 h in 50 mM phosphate buffer (pH 7.0). The samples were cooled on ice for 30 min and the residual inhibitory activity against each enzyme was determined as described above. All the assays were performed in triplicate and the mean and SD values were calculated.

### Structural analysis of fowlerstefin

To analyze the native molecular size and structure of fowlerstefin, gel filtration chromatography was performed with a Superdex 200 HR 10/30 column using an Äcta FPLC system (GE Biosciences, Pittsburgh, PA, USA). Purified recombinant fowlerstefin (1 mg) was loaded onto the column and fractions (0.5 ml each) were collected. The collected fractions were separated *via* SDS-PAGE and their inhibitory activities against NfCPB-L were determined. The column was calibrated with the following molecular weight markers (Sigma-Aldrich): blue dextran (2000 kDa), β-amylase (200 kDa), alcohol dehydrogenase (150 kDa), BSA (66 kDa), carbonic anhydrase (29 kDa), and cytochrome *c* (12.4 kDa). The *K*_av_ value of each protein was calculated based on the equation *K*_av_ = (*V*_e_ − *V*_0_)/(*V*_t_ − *V*_0_), where *V*_e_ is the elution volume of the protein, *V*_0_ denotes the elution volume of blue dextran and *V*_t_ refers to the total bed volume. The molecular structure of fowlerstefin was further analyzed using electrophoretic methods. Purified recombinant fowlerstefin (20 μg) was electrophoresed in the presence and absence of SDS without heating as described previously [25]. The samples treated as above were analyzed *via* SDS-PAGE followed by Coomassie blue staining.

### Expression profile of fowlerstefin at different developmental stages of *N. fowleri*

Encystation of *N. fowleri* trophozoites was induced by incubating the amoebae in encystment medium (120 mM NaCl, 0.03 mM MgCl_2_, 1 mM NaHPO_4_, 1 mM KH_2_PO_4_, 0.03 mM CaCl_2_, 0.02 mM FeCl_2_, pH 6.8) [26]. In brief, *N. fowleri* trophozoites (approximately 2 × 10^5^ cells) were washed with PBS (pH 7.4) three times and incubated in 6-well plates with 5 ml of encystment medium at 37 °C for 0, 6, 12, 24, 36, 48 or 72 h. The morphological changes of the amoebae at each indicated time were determined using an EVOS® XL Core microscope (Life Technologies, Carlsbad, California, USA). At each time point, amoeba cells were collected, rinsed with PBS several times and the total RNAs were isolated by using TRIzol reagent (Invitrogen) according to the manufacturer’s protocols. The purified total RNA was treated with RNase-free DNase (Gibco) to remove any contaminating DNA. The cDNAs were synthesized from equal amounts of total RNA (1 μg each) using a RNA to cDNA EcoDry Premix Kit (Clontech) according to the manufacturer’s instructions. Reverse transcription PCR (RT-PCR) was performed using specific primers for fowlerstefin, NfCPB and NfCPB-L. Glyceraldehyde 3-phosphate dehydrogenase of *N. fowleri* (NfGAPDH) was also included as an internal control. The amplified PCR products were analyzed on a 1.5% agarose gel, stained with RedSafe™ Nucleic Acid Staining Solution (Intron Biotechnology, Seongnam, Korea) and visualized under ultraviolet (UV) light. ImageJ (https://imagej.nih.gov/ij/) was used for densitometric analysis. Expression of fowlerstefin was also analyzed by immunoblotting. The lysates of *N. foweleri* trophozoites and cysts were prepared by repeated freezing-thawing in RIPA Lysis and Extraction buffer (Thermo Fisher Scientific*)* followed by sonication on ice. The lysate of *N. foweleri* trophozoites (20 μg) and cysts (20 μg) were separated by 15% SDS-PAGE and transferred electrophoretically onto the nitrocellulose membrane. The membrane was blocked with PBS supplemented with 0.05 Tween 20 (PBST) and 5% skim milk for 1 h and incubated with anti-fowlerstefin (1:1000 dilution in 5% skim milk) at room temperature for 2 h. After several washes with PBST, the membrane was incubated with horseradish peroxidase (HRP)-conjugated anti mouse IgG (Sigma-Aldrich) (1:1000 dilution in 5% skim milk) at room temperature for 2 h. The membrane was washed with PBST several times and immune-reactive bands were detected using the enhanced chemiluminescence (ECL) substrate (Thermo Fisher Scientific).

### Localization of fowlerstefin

To analyze the localization of fowlerstefin in the amoebae, immunoblot analysis was performed. The ESP was prepared by incubating *N. fowleri* trophozoites in PBS for 1 h at 37 °C. After centrifugation at 800×*g* for 5 min, the supernatant was collected, concentrated and used as the ESP. The *N. fowleri* lysate was prepared *via* repeated freezing-thawing of *N. fowleri* trophozoites. The sample was centrifuged at 20,000×*g* for 20 min at 4 °C and the supernatant was collected and used as the *N. fowleri* lysate. The *N. fowleri* ESP (20 μg) and lysate (20 μg) were separated by SDS-PAGE and transferred electrophoretically onto the nitrocellulose membrane. Immunoblot was carried out with the same protocols described above.

### BV-2 cell culture and treatment with recombinant fowlerstefin

The mouse microglia cell line BV-2 was maintained in Dulbecco’s modified Eagle’s medium (DMEM; Welgene, Daegu, Korea) [17]. The experiment was performed by seeding the cells on 6-well dishes (2 × 10^5^ cells/well). Cells were cultured to approximately 70% confluence and a fresh serum-free medium was added for 12 h before lipopolysaccharide (LPS) or fowlerstefin treatment. To determine the non-lethal amounts of fowlerstefin for BV-2 microglial cells, the cell cytotoxicity of different amounts of fowlerstefin was assessed using the CytoTox 96® Non-radioactive cytotoxicity assay kit (Promega, Madison, WI, USA). BV-2 cells (2 × 10^5^ cells/well) cultured in DMEM supplemented with 10% FBS in a 96-well microplate were treated with different concentrations of fowlerstefin (0 to 50 µg/ml) at 37 °C for 24 h. The supernatant was drained from the plate and 50 µl of CytoTox 96® reagent was added to each well. Subsequently, 50 µl of stop solution was added to each well and the reaction was read at 490 nm with a Multiskan FC microplate reader (Thermo Fisher Scientific). No cellular damage was observed when the cells were treated with up to 20 µg/ml of fowlerstefin (Additional file 2: Figure S2) and a final concentration of fowlerstefin (15 µg/ml) was selected for further analysis.

### Cytokine array assay

The overall expression profile of diverse cytokines and chemokines in BV-2 cells stimulated with fowlerstefin was analyzed. BV-2 cells were treated with fowlerstefin (15 µg/ml) or PBS for 9 h. The cell culture supernatant was collected by centrifugation at 1000×*g* for 5 min at 4 °C and analyzed with the Proteome Profiler^TM^ Mouse Cytokine Array Panel A (R&D systems, Minneapolis, Minnesota, USA) followed by the protocols provided by the manufacturer. The culture supernatant obtained from BV-2 cells treated with PBS was used as a negative control.

### RT-PCR for cytokine expressions

To analyze the effect of fowlerstefin on the expression of several major cytokines in BV-2 cell, the cells were stimulated with fowlerstefin (15 µg/ml) for varying time periods (0, 3, 6, 9 and 12 h) and harvested at the indicated time points. The cells were washed with ice-cold PBS and the total RNA was isolated using TRIzol (Invitrogen) according to the manufacturer’s instructions. The amount of total RNA was quantitated using the DeNovix DS-11 microvolume spectrophotometer (Wilmington, Delaware, USA). Total RNA (1 µg) was reverse-transcribed into cDNA using a RNA to cDNA EcoDry Premix Kit (Clontech) according to the manufacturer’s instructions. The RT reactions were performed for 1 h at 42 °C. Subsequently, PCR was performed using primer sets specific for mouse GAPDH (forward: 5ʹ-ACC ACA GTC CAT GCC ATC AC-3ʹ; reverse: 5ʹ-CAC CAC CCT GTT GCT GTA GCC-3ʹ), mouse TNF (forward: 5ʹ-CAT CTT CTC AAA ATT CGA GTG ACA A-3ʹ; reverse: 5ʹ-TGG GAG TAG ACA AGG TAG AAC CC-3ʹ), mouse IL-6 (forward: 5ʹ-CGG AGA GGA GAC TTC ACA G-3ʹ; reverse: 5ʹ-GGA AAT TGG GGT AGG AAG GA-3ʹ), mouse IL-1α (forward: 5ʹ-ATG GCC AAA GTT CCT GAC TT-3ʹ; reverse: 5ʹ-TGG TCT TCT CCT TGA GCG CT-3ʹ), mouse MIP-2 (forward: 5ʹ-CCA AGG GTT GAC TTC AAG AAC-3ʹ; reverse: 5ʹ-GCG AGG CAC ATC AGG TAC G-3ʹ) and mouse IL-1β (forward: 5ʹ-TGC AGA GTT CCC CAA CTG GTA CAT-3ʹ; reverse: 5ʹ-GTG CTG CCT AAT GTC CCC TTG AAT-3ʹ). The amplified products were separated on a 1.5% agarose gel, stained with RedSafe™ Nucleic Acid Staining Solution (Intron Biotechnology) and were observed under UV light. ImageJ (https://imagej.nih.gov/ij/) was used for densitometric analysis.

### Enzyme-linked immunosorbent assay (ELISA)

The production of IL-6 and TNF from BV-2 cells following fowlerstefin stimulation was quantified using the mouse Quantikine IL-6 and TNF ELISA kits (R&D systems). BV-2 cell were treated with fowlerstefin (15 µg/ml) for 0, 3, 6, 9 and 12 h. The culture supernatants were collected at indicated time points and the amount of IL-6 and TNF in the supernatants was measured according to the manufacturer’s protocols. BV-2 cells treated with the PBS and LPS (3 µg/ml) were used as negative and positive controls, respectively.

### Mitogen-activated protein kinase (MAPK) signaling pathway analysis

To analyze the proinflammatory signaling pathway induced by fowlerstefin, the effects of MAPK inhibitors on IL-6 and TNF productions in BV-2 cells were analyzed. Inhibitors for p38 (SB203580), c-Jun N-terminal kinase (JNK) (SP600125), extracellular signal-regulated protein kinase (ERK) (U0126), NF-κB (MG132) and AP-1 (SR11302) were used in this study. All inhibitors were purchased from Calbiochem (San Diego, California, USA). BV-2 cells were seeded in 6-well dishes (2 × 10^5^ cells/well) and cultured to approximately 70% confluence. After changing the media with fresh serum-free media, each inhibitor was added to the cells and incubated for 3 h. Fowlerstefin (15 µg/ml) was added to the cells pretreated with each inhibitor followed by incubation for an additional 3 h. BV-2 cells treated with PBS and LPS (3 µg/ml) were used as negative and positive controls, respectively. Cells treated with only fowlerstefin (15 µg/ml) were also included as a control. The cells were washed with ice-cold PBS and the total RNA was isolated using the same protocols described above and reverse-transcribed into cDNA. Changes in IL-6 and TNF expression in the cells were analyzed *via* PCR as described above. ImageJ (https://imagej.nih.gov/ij/) was used for densitometric analysis. Production of IL-6 and TNF was also comparatively analyzed using the mouse Quantikine IL-6 and TNF ELISA kits (R&D systems).

### Statistical analysis

Data were represented as the mean ± SD of three individual assays. Statistical significance was determined by one-way analysis of variance (ANOVA), followed by Dunnett’s post hoc test comparing all the concentrations with the control using GraphPad Prism 7 (GraphPad Software, San Diego, CA). Differences in mean values were considered statistically significant when the *P-*value was less than 0.05.

## Results

### Sequence and phylogenetic analyses of fowlerstefin

**Fig. 1:**
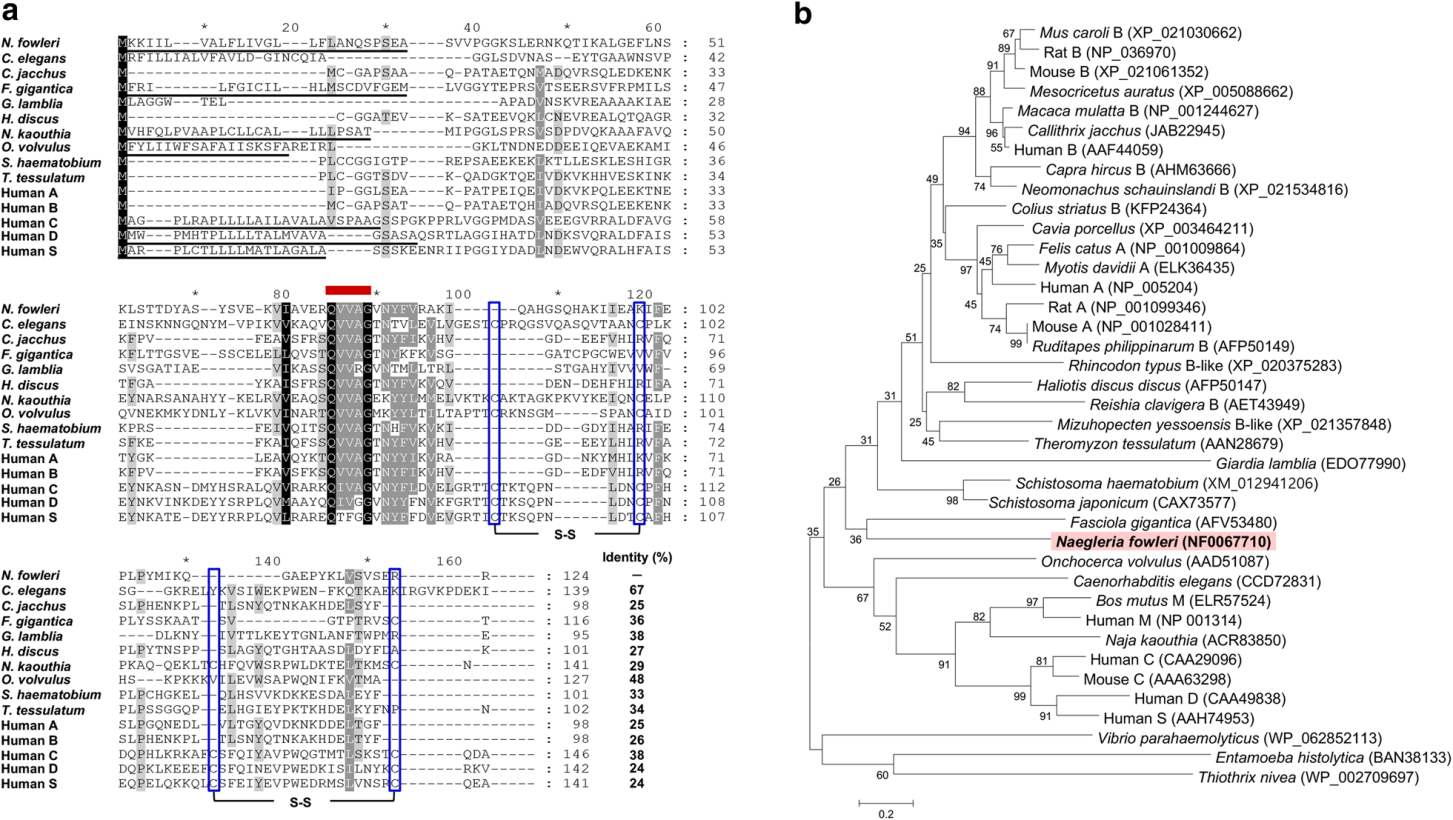
Multiple sequence alignment and phylogenetic analysis. **a** Multiple sequence alignment. The deduced amino acid sequence of fowlerstefin was aligned with sequences of related proteins from other parasites and humans. Gaps are introduced into the sequences to maximize the alignment. The QVVAG cystatin motif is indicated as a red bold line above the sequence. The predicted N-terminal signal peptide sequences are underlined. No putative N-glycosylation site was identified in the fowlerstefin sequence. Potential disulfide bridges are shown with brackets, while cysteine residues are boxed. **b** Phylogenetic analysis. The tree was built by the neighbor-joining method using MEGA 4. Numbers at the branches indicate bootstrap proportions (1000 replicates)

The gene of fowlerstefin consisted of 375 bp, encoding 124 amino acid residues with a predicted molecular mass of about 13.7 kDa. A primary sequence analysis of fowlerstefin revealed a putative N-terminal signal peptide sequence of 26 amino acid residues. The sequence analysis of fowlerstefin indicated a highly conserved Gln-Val-Val-Ala-Gly (QVVAG) motif in the middle of the sequence (Fig. 1a). Fowlerstefin lacks the C-terminal cysteine residues, which are involved in disulfide bond formation in stefin C, D and S super-family [27, 28]. Phylogenetic analysis showed that fowlerstefin was clustered into family A or B of the stefin family (Fig. 1b).

### Production and purification of recombinant fowlerstefin

**Fig. 2:**
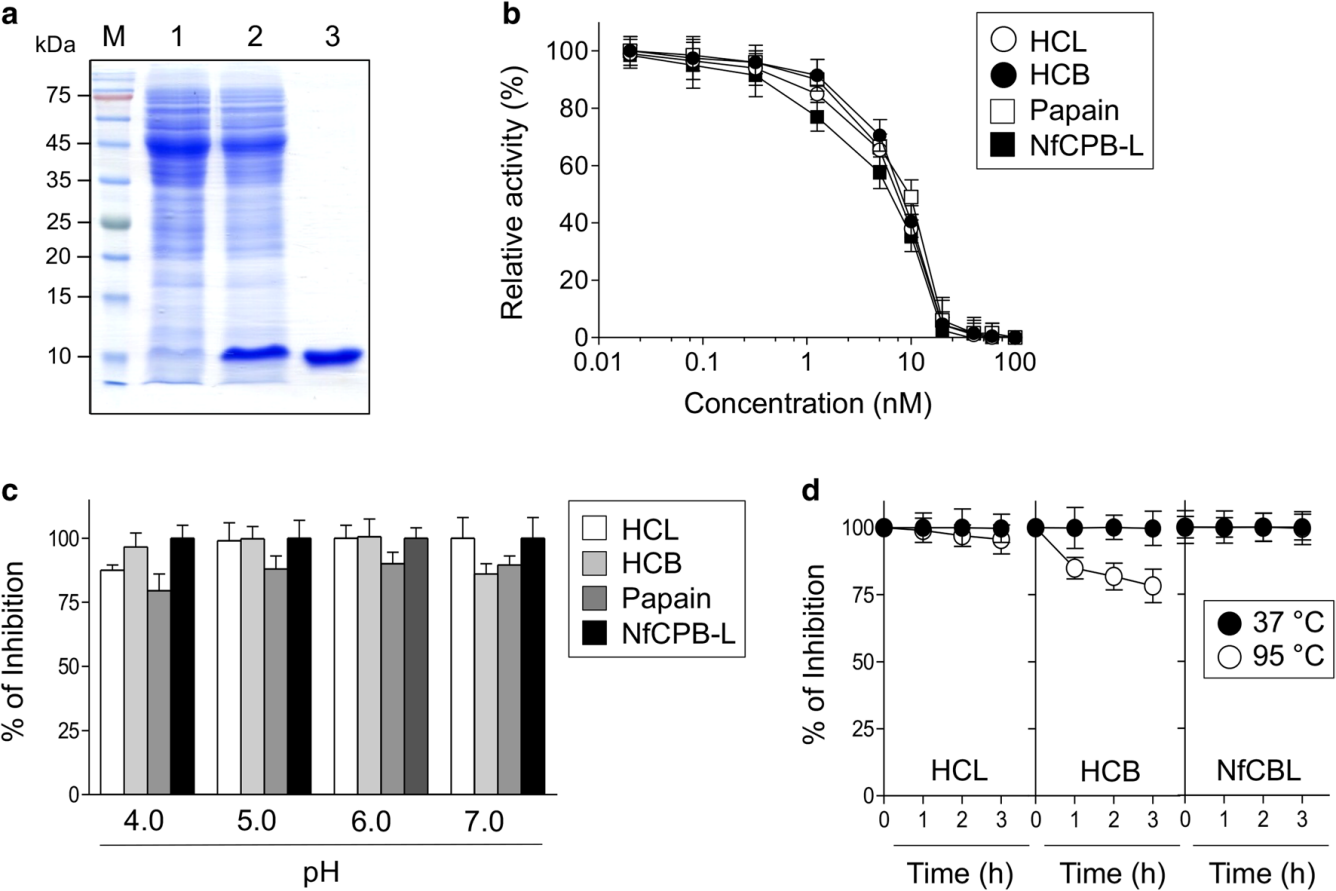
Production and characterization of recombinant fowlerstefin. **a** Expression and purification of recombinant fowlerstefin. The recombinant fowlerstefin was purified with Ni-NTA affinity chromatography and analyzed by SDS-PAGE. Lane 1: non-induced *E. coli* lysate (20 µg); Lane 2: IPTG-induced *E. coli* lysate (20 µg); Lane 3: Ni-NTA affinity purified fowlerstefin (10 µg). **b** Dose-dependent inhibition assay. Inhibitory activity of fowlerstefin against human cathepsin B (HCB), human cathepsin L (HCL), papain and NfCPB-L was analyzed. Each individual enzyme (20 nM) was incubated with different concentrations of fowlerstefin and the residual enzyme activity was measured. The assay buffers for each enzyme were as follows: HCB and HCL, 50 mM sodium acetate (pH 6.0); papain, 50 mM sodium acetate (pH 5.0); and NfCPB-L, 50 mM sodium acetate (pH 4.5). Results are expressed as the percentage of inhibited enzyme activity compared to control without fowlerstefin. Three independent assays were performed for each enzyme and mean and standard deviation (SD) were calculated. **c** Inhibitory activity of fowlerstefin. Fowlerstefin was incubated with HCL, HCB, papain or NfCPB-L in different pH buffers for 30 min at room temperature, after which the residual enzyme activity of each enzyme was assayed. All experiments were performed in triplicate and the mean and SD values were represented. **d** Thermal stability. Fowlerstefin was incubated in 50 mM sodium phosphate (pH 7.0) at 37 °C or 95 °C for the indicated times, after which the residual inhibitory activity for HCL, HCB and NfCPB-L was analyzed. All experiments were performed in triplicate and the mean and SD values were calculated

The recombinant fowlerstefin was expressed in *E. coli* as a soluble form. The purified recombinant protein had an apparent molecular mass of 11 kDa (Fig. 2a), which was matched with the predicted molecular mass of the deduced amino acid sequence of fowlerstefin.

### Inhibitory activity of fowlerstefin against cysteine proteases

The inhibitory activity of fowlerstefin against several cysteine proteases, including HCB, HCL, papain and NfCPB-L, was investigated. Fowlerstefin effectively inhibited the enzymatic activities of all tested enzymes with a dose-dependent manner (Fig. 2b). Fowlerstefin effectively inhibited all of the tested enzymes over a wide range of pH values (Fig. 2c). Fowlerstefin was highly stable at 37 °C. It also maintained its inhibitory activity up to 75% upon exposure to a temperature of 95 °C for 3 h (Fig. 2d).

### Monomeric structure of fowlerstefin

**Fig. 3:**
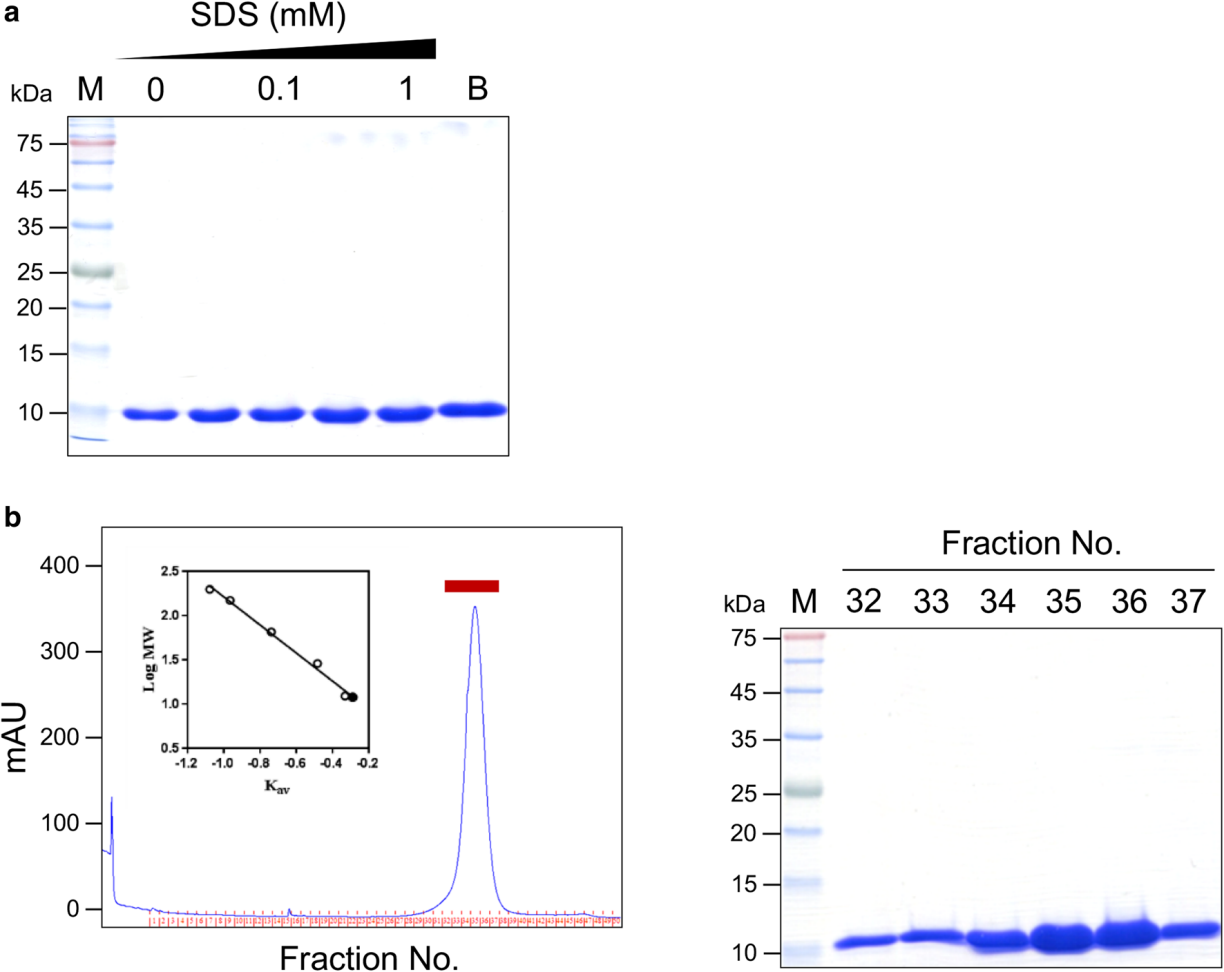
Structure analysis of fowlerstefin. **a** Effect of SDS on fowlerpain structure. Fowlerstefin was incubated in different concentration of SDS (0–1 mM) at room temperature and separated by SDS-PAGE without heating. Lane M: siz parker proteins; Lane B: boiled sample in the presence of both SDS and β-ME. **b** Gel filtration chromatography. The *K*_av_ value of fowlerstefin (closed circles) was calculated and compared to those of standard marker proteins (open circles). The fractions with strong inhibitory activity against NfCPB-L (fractions 32 to 37, red bar) were further analyzed by SDS-PAGE. Lane M: size marker proteins

SDS-PAGE analysis of fowlerstefin without prior heating in the presence of different concentrations of SDS suggested a monomeric structure (Fig. 3a). Gel filtration chromatography was performed to further analyze the native molecular mass of fowlerstefin (Fig. 3b). Each fraction was analyzed by measuring its inhibitory activity against NfCPB-L. Fractions 32 to 37 showed strong inhibitory activity against NfCPB-L. Fowlerstefin was identified in fractions 32 to 37 and its native molecular mass was estimated at approximately 11 kDa. These data indicated that fowlerstefin carried a monomeric structure and was functional as a monomer.

### Expression profile of fowlerstefin in different developmental stages of *N. fowleri*

**Fig. 4:**
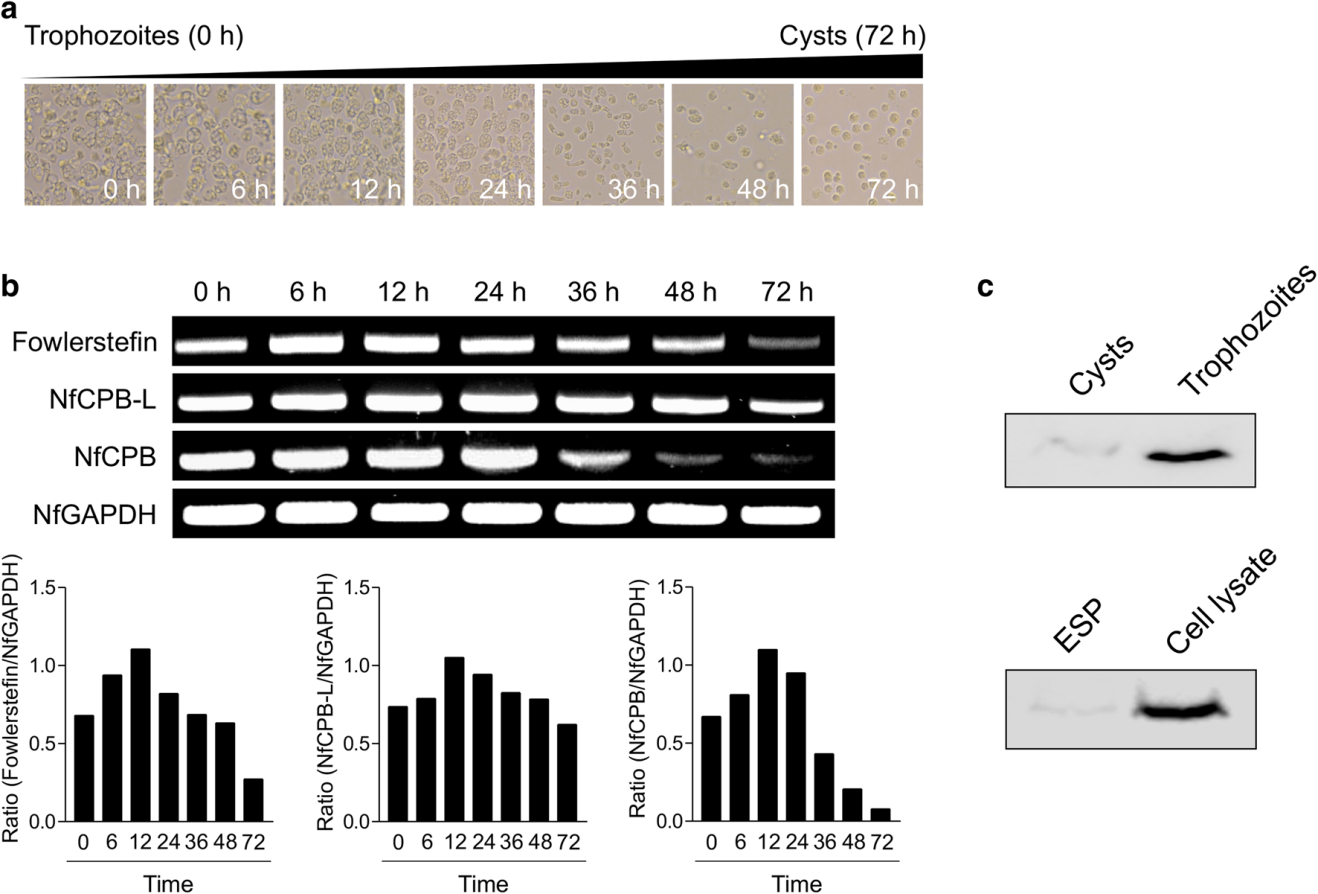
Expression profile of fowlerstefin. **a** Morphological changes. Morphological changes of *N. fowleri* trophozoites into precysts (or cysts) cultured in encystation media for 0, 6, 12, 24, 36, 48 and 72 h. **b** RT-PCR analysis. The expression profiles of fowlerstefin, NfCPB, NfCPB-L and NfGAPDH were analyzed in different developmental stages of *N. fowleri*. Graphs show the densitometric ratios of fowlerstefin, NfCPB and NfCPB-L to NfGAPDH. **c** Immunoblot analysis. Expression pattern of fowlerstefin in cysts and trophozoites was analyzed by immunoblot with anti-fowlerstefin (upper blot). The ESP and cell lysates of *N. fowleri* trophozoies were analyzed by immnoblot with anti-fowlerstefin (lower blot)

Trophozoites of *N. fowleri* cultured in encystment medium started morphological changes after 6 h incubation. Precysts were observed after 24 h incubation and most *N. fowleri* trophozoites transformed into cysts after 72 h incubation (Fig. 4a). The expression profile of fowlerstefin in the different developmental stages of *N. fowleri* was analyzed *via* semi-quantitative RT-PCR. Fowlerstefin was clearly expressed in both trophozoite and cyst stages; however, the transcription level decreased remarkably from trophozoites to cysts (Fig. 4b). The two cathepsin B cysteine proteases of *N. fowleri*, NfCPB and NfCPB-L, also showed similar patterns of expression in different developmental stages of *N. fowleri*. The NfGAPDH, which was included as the internal control, showed a constant level of expression throughout all the tested developmental stages of *N. fowleri*. Immunoblot analysis with anti-fowlerstefin also suggested that this protein was predominantly expressed in the trophozoite stage (Fig. 4c). Fowlerstefin was mainly identified in the cell lysate of the amoebae, although a weak immunopositive reaction was also observed in the ESP (Fig. 4c).

### Cytokine expression profile of BV-2 microglial cells stimulated by fowlerstefin

**Fig. 5:**
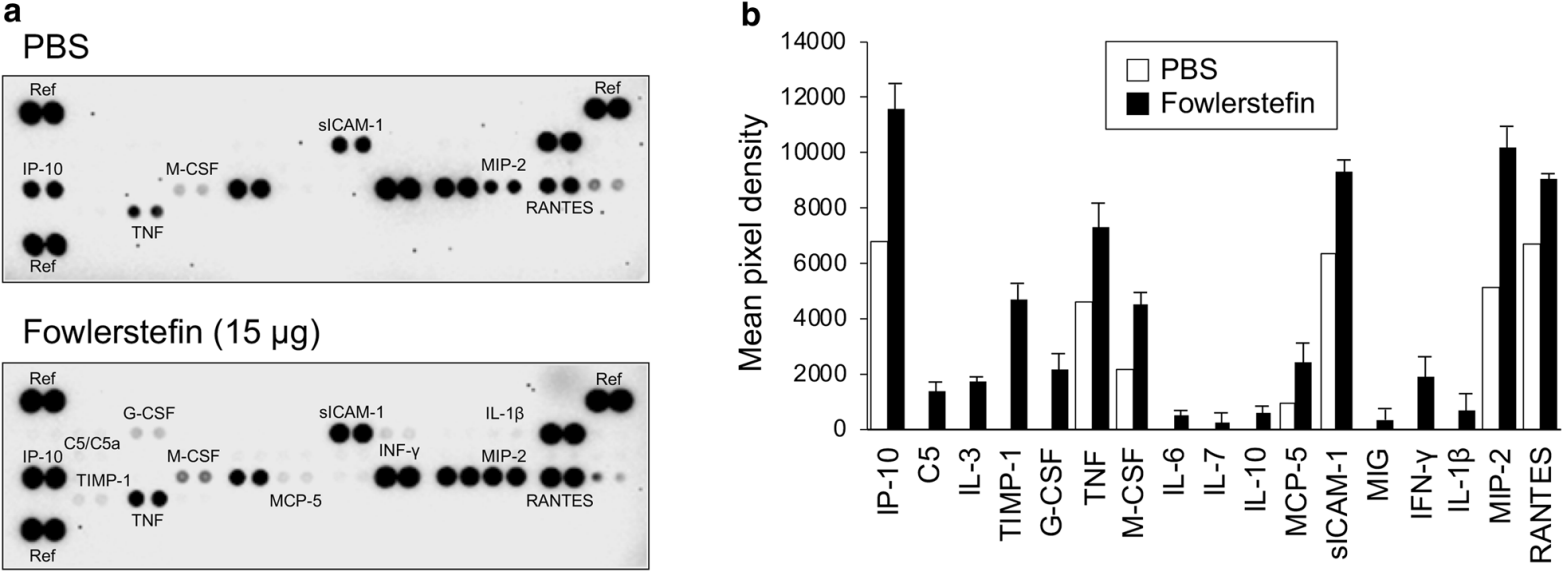
Cytokine profile analysis of BV-2 cells stimulated by fowlerstefin. **a** Cytokine array assay. BV-2 cells were stimulated with fowlerstefin (15 µg/ml) for 9 h and the culture supernatants were collected and subjected to cytokine array analysis. Culture supernatant from PBS treated cells was used as a negative control. The image shows the results of one of two independent experiments which revealed similar expression patterns. **b** Quantitative analysis of mean density from the cytokine array assay. Data are the mean vaules from two independent experiments

**Fig. 6:**
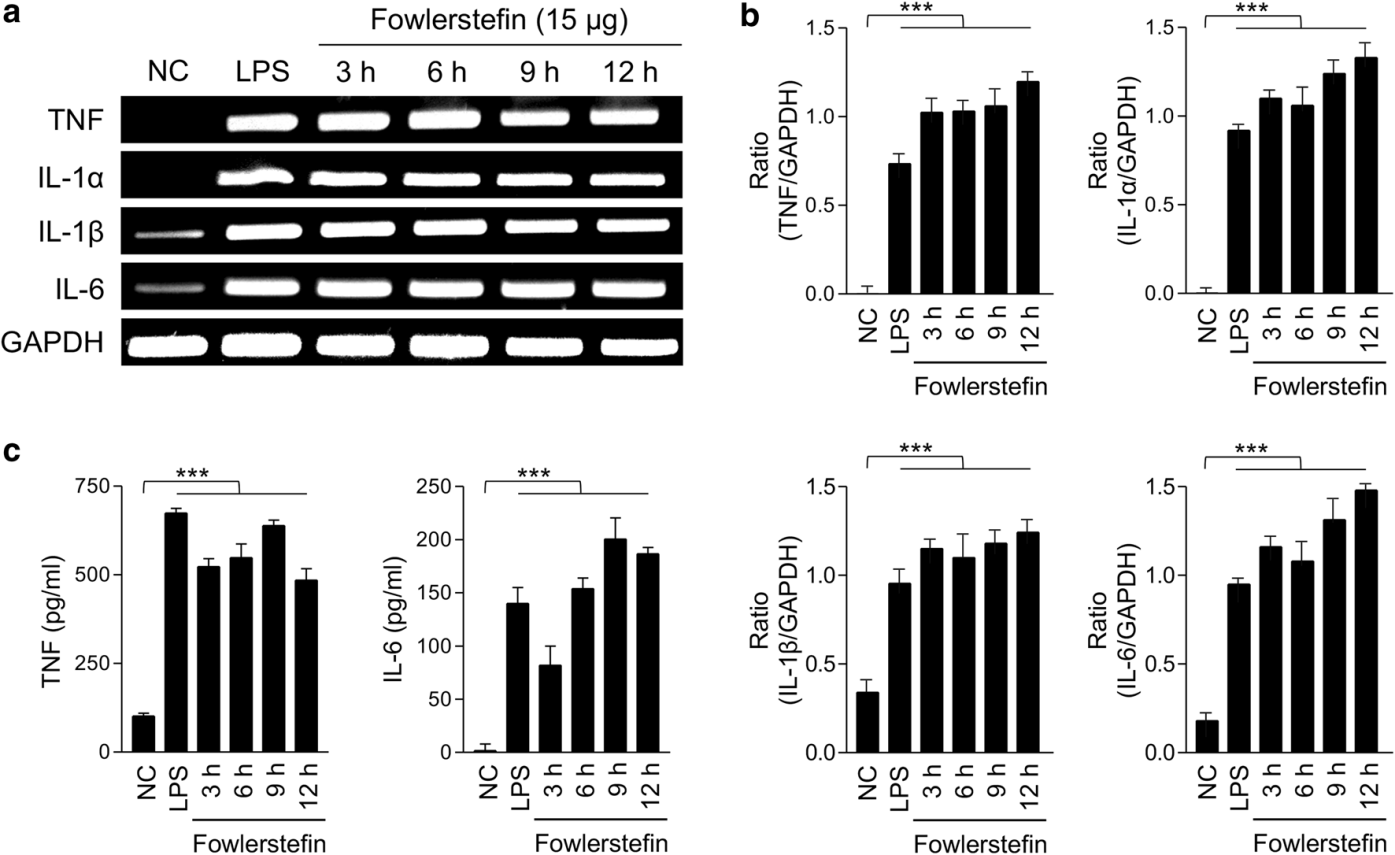
Fowlerstefin induces TNF, IL-1α, IL-1β and IL-6 expression in BV-2 cells. **a** RT-PCR analysis of TNF, IL-1α, IL-1β and IL-6 mRNA expression in BV-2 cells. BV-2 cells were treated with fowlerstefin (15 µg/ml) and harvested after 0–12 h. Total RNA was isolated from the cells and the expression of TNF, IL-1α, IL-1β and IL-6 was assessed by RT-PCR. GAPDH was used as an internal control. Lane NC: control not treated with fowlerstefin; Lane LPS: LPS (3 µg/ml); Lanes 3, 6, 9 and 12: treated with fowlerstefin for the indicated times. LPS was treated with the cells for 2 h. A representative gel image of three independent experiments which revealed similar patterns is presented. **b** Quantitative analysis of TNF, IL-1α, IL-1β and IL-6 relative to GAPDH. Graphs show the mean ± SD densitometric ratios of cytokines to GAPDH of three independent experiments. **c** ELISA. BV-2 cells were treated with fowlerstefin (15 µg/ml) for 0–12 h. At various time points during the incubation, the culture supernatant was collected and the levels of TNF, IL-1α, IL-1β and IL-6 were analyzed by ELISA. Values are presented as the mean ± SD of three independent experiments. One-way ANOVA with Dunnett’s *post-hoc* test was performed as multiple comparisons with the control (NC) (for details, see Additional file 3: Text S1). ***P < 0.0001

Fowlerstefin induced the expression of cytokines and chemokines in BV-2 microglial cells (Fig. 5). The expression level of diverse cytokines and chemokines including TNF, IP-10, M-CSF, sICAM, MIP-2 and RANTES was strongly increased in BV-2 cells stimulated with fowlerstefin. The expression of C5, IL-5, TIMP-1, G-CSF, IL-6, MCP-3, MIG, IFN-γ and IL-1β was also increased in BV-2 cells upon treatment with fowlerstefin. These results suggested that fowlerstefin induced the expression of diverse cytokines and chemokines in BV-2 microglial cells, which are associated with the pro-inflammatory response. RT-PCR was performed to confirm the elevated transcription of the cytokines in BV-2 cells treated with fowlerstefin. Consistent with the results of the cytokine array assay, the expression of several cytokines, including TNF, IL-1α, IL-1β and IL-6, was increased in BV-2 cells stimulated with fowlerstefin (Fig. 6a, b). The expression of all cytokines reached the highest level at 12 h after treatment of fowlerstefin; TNF (ANOVA: *F*_(5, 12)_ = 152, *P* < 0.0001), IL-1α (ANOVA: *F*_(5, 12)_ = 234, *P* < 0.0001), IL-1β (ANOVA: *F*_(5, 12)_ = 30.9, *P* < 0.0001) and IL-6 (ANOVA: *F*_(5, 12)_ = 155, *P* < 0.0001) (Additional file 3: Text S1). Upregulation of TNF and IL-6 protein productions was further analyzed by ELISA. Significant increase of the levels of these cytokines was identified in BV-2 microglial cells following treatment with fowlerstefin; TNF (ANOVA: *F*_(5, 12)_ = 209, *P* < 0.0001) and IL-6 (ANOVA: *F*_(5, 12)_ = 178, *P* < 0.0001) (Fig. 6c).

### Fowlerstefin-induced TNF and IL-6 production is mediated by MAPK activation

**Fig. 7:**
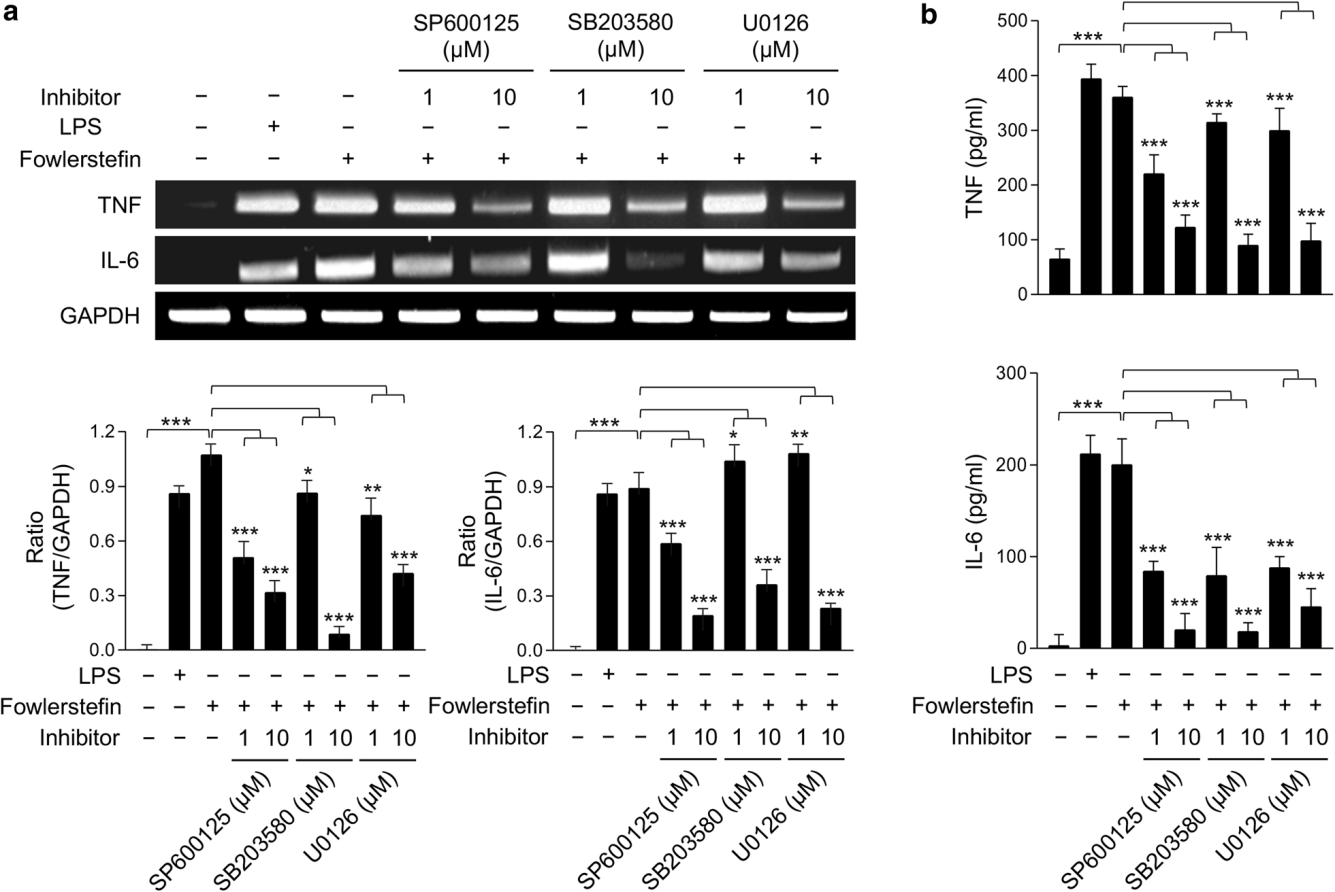
Effect of JNK, p38 and ERK inhibitors on the expression of TNF and IL-6 in BV-2 cells stimulated with fowlerstefin. **a** RT-PCR analysis. BV-2 cells were pretreated with different concentrations (0, 1, or 10 µM) of JNK inhibitor (SP600125), p38 inhibitor (SB203580) or ERK inhibitor (U0126) for 3 h before the cells were incubated with fowlerstefin (15 µg/ml) for 3 h. The cells were harvested and the expression of TNF and IL-6 was analyzed by RT-PCR. GAPDH was used as an internal control. A representative gel image of three independent experiments which revealed similar patterns is presented. Graphs show the mean ± SD densitometric ratios of TNF and IL-6 to GAPDH of three independent experiments. **b** ELISA. The amount of TNF and IL-6 expressed by the BV-2 cells was quantitatively measured by ELISA. Values are presented as the mean ± SD of three independent experiments. One-way ANOVA with Dunnett’s *post-hoc* test was performed to determine significant differences from the inhibitor-untreated cells (for details, see Additional file 3: Text S1). *P < 0.05, **P < 0.001, ***P < 0.0001

To understand the signaling pathways mediating fowlerstefin-induced IL-6 and TNF production in BV-2 microglial cells, studies using MAPK inhibitors were performed. Treatment of BV-2 microglial cells with p38 inhibitor (SB203580), JNK inhibitor (SP600125) and ERK inhibitor (U0126) prior to fowlerstefin stimulation effectively downregulated the mRNA expression of TNF and IL-6 in a dose-dependent manner (Fig. 7a). ELISA analysis was also conducted to analyze the changes in TNF and IL-6 production in BV-2 cells pretreatment with each MAPK inhibitor. Fowlerstefin-induced TNF and IL-6 production in BV-2 cells was reduced following pretreatment with p38, JNK and ERK inhibitors in a dose-dependent manner; TNF (ANOVA: *F*_(8, 18)_ = 790, *P* < 0.0001) and IL-6 (ANOVA: *F*_(8, 18)_ = 1826, *P* < 0.0001) (Fig. 7b, Additional file 3: Text S1). These results collectively suggested that the fowlerstefin-induced pro-inflammatory response in BV-2 cells was mediated *via* MAPK signaling pathway.

### Fowlerstefin-induced TNF and IL-6 production is regulated *via* NF-κB and AP-1 signaling pathways

**Fig. 8:**
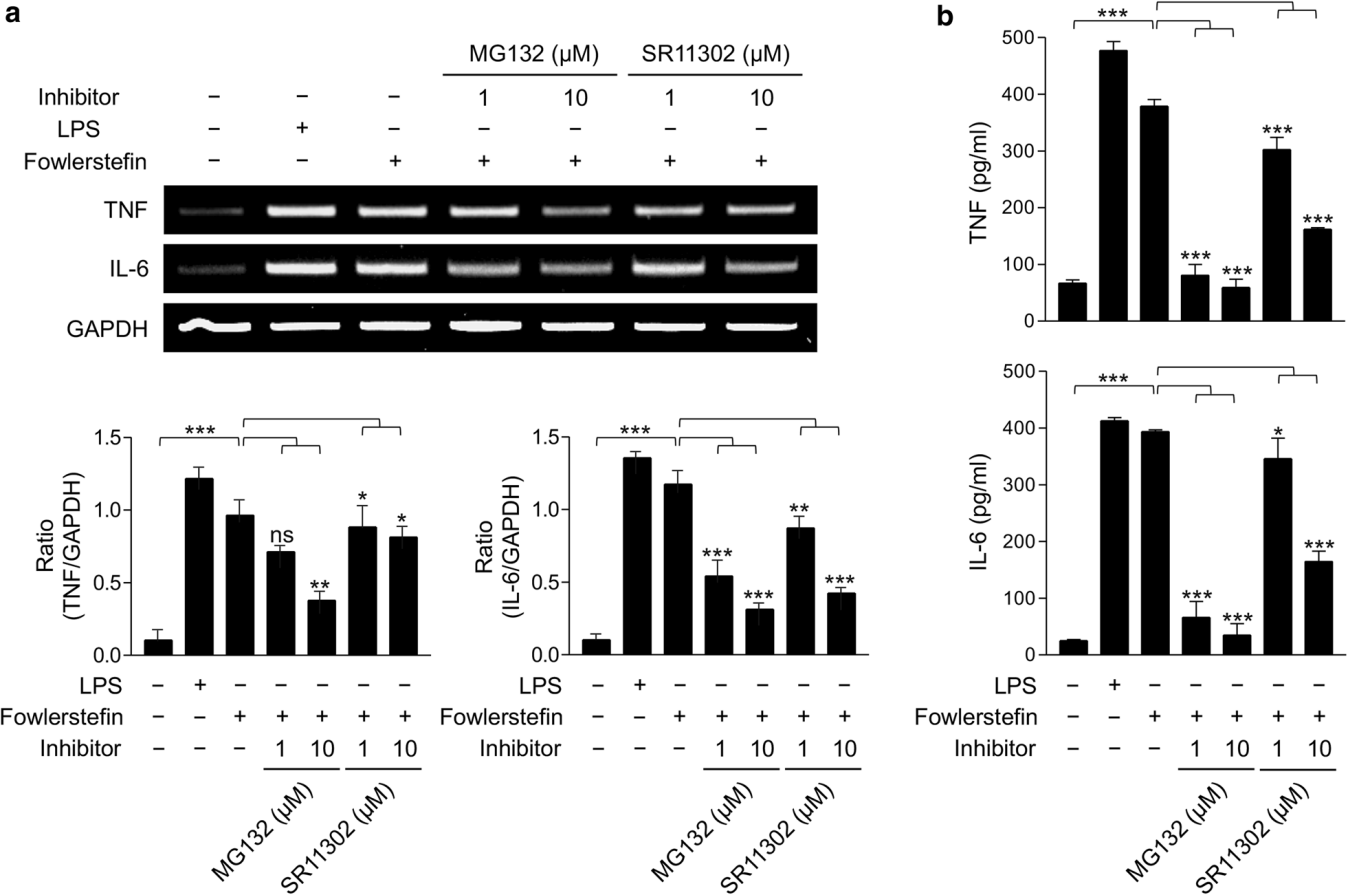
Effect of NF-κB and AP-1 inhibitors on the expression of TNF and IL-6 in BV-2 cells stimulated with fowlerstefin. **a** BV-2 cells were pretreated with different concentrations (0, 1, or 10 µM) of NF-κB inhibitor (MG132) or AP-1 inhibitor (SR11302) for 3 h and the cells were then incubated with fowlerstefin (15 µg/ml) for 3 h. The expression of TNF and IL-6 transcripts was analyzed by RT-PCR. GAPDH was used as an internal control. A representative gel image of three independent experiments which revealed similar patterns is presented. Graphs show the mean ± SD densitometric ratios of TNF and IL-6 to GAPDH of three independent experiments. **b** ELISA. Production of TNF and IL-6 was quantitatively measured by ELISA. Values are presented as the mean ± SD of three independent experiments. One-way ANOVA with Dunnett’s *post-hoc* test was performed to determine significant differences from the inhibitor-untreated cells (for details, see Additional file 3: Text S1). ns, not significant, *P < 0.05, **P < 0.001, ***P < 0.0001

Treatment of BV-2 cells with NF-κB inhibitor (MG132) and AP-1 inhibitor (SR11302) prior to fowlerstefin treatment resulted in a reduced expression of TNF and IL-6 in a dose-dependent manner (Fig. 8a). The expression of both TNF and IL-6 was downregulated by MG132 and SR11302, even though they were not greatly affected by the low concentration of SR11302. The influence of of NF-κB and AP-1 inhibitors on the expression of TNF and IL-6 was also confirmed by ELISA. Consistent with the RT-PCR results, the expression of TNF and IL-6 in BV-2 cells induced by fowlerstefin was highly inhibited by MG132: TNF (ANOVA: *F*_(6,14)_ = 3049, *P* < 0.0001) and IL-6 (ANOVA: *F*_(6,14)_ = 338, *P* < 0.0001) (Fig. 8b, Additional file 3: Text S1).

## Discussion

A novel cysteine protease inhibitor of *N. fowleri*, known as fowlerstefin, has been characterized. Fowlerstefin is a typical cysteine protease inhibitor belonging to the stefin A or B cystatin superfamily. The QVVAG motif, a characteristic element that is tightly conserved in the cystatin family proteins and plays a key role in the direct interactions with the target enzyme [27, 28], was found in the middle region of fowlerstefin. Although fowlerstefin has the putative signal peptide sequences in its N-terminal region, the C-terminal cysteine residues, which are crucial for disulfide bond formation in extracellular cystatins [28, 29], were absent. Immunoblot analysis of ESP also provided evidence that fowlerstefin might not be an actively secreted protein. Fowlerstefin effectively inhibited cysteine proteases such as HCB, HCL, papain and NfCPB-L. Heat or pH did not affect the inhibitory activity of fowlerstefin, indicating that fowlerstefin is a highly stable molecule similar to other stefin A and B superfamily proteins [30–32]. Gel filtration chromatography and SDS-PAGE analysis in the presence or absence of SDS also suggest that fowlerstefin is functional as a monomeric structure. These findings suggest that fowlerstefin is a typical stefin with structural and biochemical properties similar to stefin A or B superfamily proteins. Fowlerstefin is expressed in both trophozoites and cysts of *N. fowleri*; however, its transcription is gradually decreased from trophozoites to cysts, which coincides with the expression profiles of NfCPB and NfCPB-L. The two enzymes are major lysosomal cysteine proteases of *N. fowleri* and are probably associated with the pathogenicity of the amoeba *via* attachment to the host tissue, evasion of the host immune system and nutrient uptake [23]. The expression patterns of fowlerstefin and the two NfCPBs suggest that fowlerstefin may be an endogenous regulator that modulates the activity and maturation of the enzymes in the amoeba. However, more comprehensive studies investigating fowlerstefin-NfCPBs interactions are required to elucidate the regulatory mechanism and the biological function of fowlerstefin.

Microglia are macrophage-like cells found in brain. They act as the major inflammatory cell types in the brain and respond to pathogens and injury by rapidly changing their morphology, proliferation and migration to the site of infection/injury where they phagocytose and destroy pathogens as well as remove damaged cells [33–35]. It has been proposed that microglia play an important role in immune modulation during *N. fowleri* infection and represent a first line of defense against CNS invasion [9]. Until now, the functional relevance of microglial cells with *N. fowleri* infection has been investigated. Trophozoites of *N. fowleri* induce the production of several pro-inflammatory cytokines such as IL-1α, IL-1β, IL-6 and TNF in microglial cells [14, 36]. *N. fowleri* lysates induce IL-1β and IL-6 production in astrocytes or rat microglial cells [18, 36]. The ESP of *N. fowleri* stimulates BV-2 microglial cells to produce IL-1α and TNF *via* NF-κB- and AP-1-dependent MAPK signaling pathways [17]. These findings suggest that microglial cells play important functions against *N. fowleri* infection and this amoeba also elicits an inflammatory response in microglial cells. In the present study, the inflammatory response of BV-2 microglial cells induced by fowlerstefin was analyzed to elucidate the mechanism underlying the immune response in the CNS as well as the patho-immunological role of fowlerstefin in *N. fowleri* infection. The cytokine array results suggest that fowlerstefin induces the secretion of several pro-inflammatory cytokines and chemokines in BV-2 microglial cells. Particularly, the expression of IL-6 and TNF, which play key role in orchestrating the inflammation responses, was increased. Inhibition of ERK, JNK and p38 greatly suppresses the expression of TNF and IL-6 in BV-2 cells both at mRNA and protein levels, which indicates that MAPKs play key regulatory functions in the production of pro-inflammatory cytokines and downstream signaling events, which lead to inflammatory responses in BV-2 microglial cells against fowlerstefin. Fowlerstefin-induced IL-6 and TNF expression is also greatly reduced by NF-κB and AP-1 inhibitors. These results suggest that fowlerstefin induces a primary inflammatory response in BV-2 microglial cells *via* NF-κB- and AP-1-dependent MAPK signaling pathways.

It is not clearly understood whether activation of microglial cells results in harmful or beneficial effects following CNS infection by microbial pathogens. A pro-inflammatory response primed by microglial cells might be crucial for the initiation of a protective immune response to infection; however, excessively activated microglial cells can induce neuronal toxicity and apoptotic cell death, a major pathologic event accompanying bacterial meningitis [37, 38]. Direct mechanical damage of neuronal cells and brain tissue by phagocytic *N. fowleri* is likely to be the main factor underlying the pathologenesis of PAM. Molecules secreted by *N. fowleri* may also hasten neuronal cell death or damage *via* indirect cytolytic activity. The secreted cytotoxic molecules, in concert with cellular debris originated from the lysed brain cells, may also attract microglial cells to the focal sites of *N. fowleri* infection [17, 36]. Fowlerstefin stimulates BV-2 microglial cells to produce pro-inflammatory cytokines including IL-6 and TNF. Considering the low levels of fowlerstefin detected in *N. fowleri* ESP, fowlerstefin is likely to be a protein that is not actively secreted or released from intact *N. fowleri* trophozoites. Instead, this protein appears to be mainly localized to the lysosomal compartments of *N. fowleri*, where it modulates the activities of endogenous cysteine proteases including NfCPBs. However, it can be released during exocytosis, along with the digested wastes in lysosomes into the extracellular space. Otherwise, fowlerstefin can be released when the amoeba is disrupted or lysed during infection. The released fowlerstefin from the amoeba encounters microglial cells and eventually stimulates a pro-inflammatory response.

## Conclusions

Identification and characterization of fowlerstefin as an endogenous cysteine protease inhibitor of *N. fowleri* provides the first insight into the regulatory mechanism of proteolysis in the amoeba. Based on the maximum expression in trophozoite stage and the consistent expression profile with NfCPBs, fowlerstefin may play a major regulatory role in modulating the activity and maturation of NfCPBs. Further in-depth studies are needed to determine the regulatory mechanism and the biological role of fowlerstefin. Fowlerstefin stimulates BV-2 microglial cells to produce pro-inflammatory cytokines including IL-6 and TNF *via* NF-κB- and AP-1-dependent MAPK signaling pathways. Fowlerstefin-induced inflammatory cytokines released from microglial cells combined with the accumulation of toxic molecules exacerbate the inflammatory response in *N. fowleri*-infected areas and generate hyperinflammation, collapse of the blood brain barrier and trigger a massive influx of peripheral immune cells from nonneuronal sites and eventually contribute to the pathogenesis of PAM. The released pro-inflammatory cytokines may also in turn activate other cell types such as astrocytes and generate a pathophysiological inflammatory cascade in the brain.

## Supplementary information

**Additional file 1: Figure S1.** Sepficity of the anti-fowlerstefin. The anti-fowlerstefin was produced in mice by pertioneal immunizations. The specificty of the anitbody was confimed against *E. coli* lysate by immunblot analysis. Lane M: protein size marker; Lane 1: non-induced *E. coli* lysate (20 µg); Lane 2: IPTG-induced *E. coli* lysate (20 µg).

**Additional file 2: Figure S2.** Cytotoxicity assay. BV-2 cells were treated with different concentrations of fowlersetfin and the cell cytotoxicity was assessed using the CytoTox 96® Non-radioactive cytotoxicity assay kit. PC: positive control represented 100% cell death. Assays were performed in triplicate and the mean and SD values were calculated. *P < 0.05.

**Additional file 3: Figure S3.** Significant results from statistical analyses in this study.

## Data Availability

The data supporting the conclusions of this article are provided within the article and its additional files. The newly generated sequence was deposited in the GenBank database under the accession number MN922292. The original datasets analysed in the present study are available from the corresponding author upon request.

## Abbreviations

ANOVA: analysis of variance
AP-1: Activator protein 1
β-ME: β-mercaptoethanol
CNS: central nervous system
ELISA: enzyme-linked immunosorbent assay
ERK: extracellular signal-regulated protein kinase
ESP: excretory and secretory proteins
GAPDH: glyceraldehyde 3-phosphate dehydrogenase
HCB: human cathepsin B
HCL: human cathepsin L
IL: interleukin
IPTG: isopropyl-1-thio-β-d-galactopyranoside
JNK: c-Jun N-terminal kinase
LPS: lipopolysaccharide
MAPK: mitogen-activated protein kinase
NfCPBs: cathepsin B-like cysteine proteases of *N. fowleri*
Ni-NTA: nickel-nitrilotriacetic acid
PAM: primary amoebic meningoencephalitis
PCR: polymerase chain reaction
RT-PCR: reverse transcription polymerase chain reaction
SDS-PAGE: sodium dodecyl sulfate-polyacrylamide gel electrophoresis
