# Supplementary figures and images for: Fowlerstefin, a cysteine protease inhibitor of Naegleria fowleri, induces inflammatory responses in BV-2 microglial cells in vitro
Source: Parasit Vectors. 2020 Jan 29;13:41. doi: 10.1186/s13071-020-3909-6 (PMC6988287; doi:10.1186/s13071-020-3909-6)

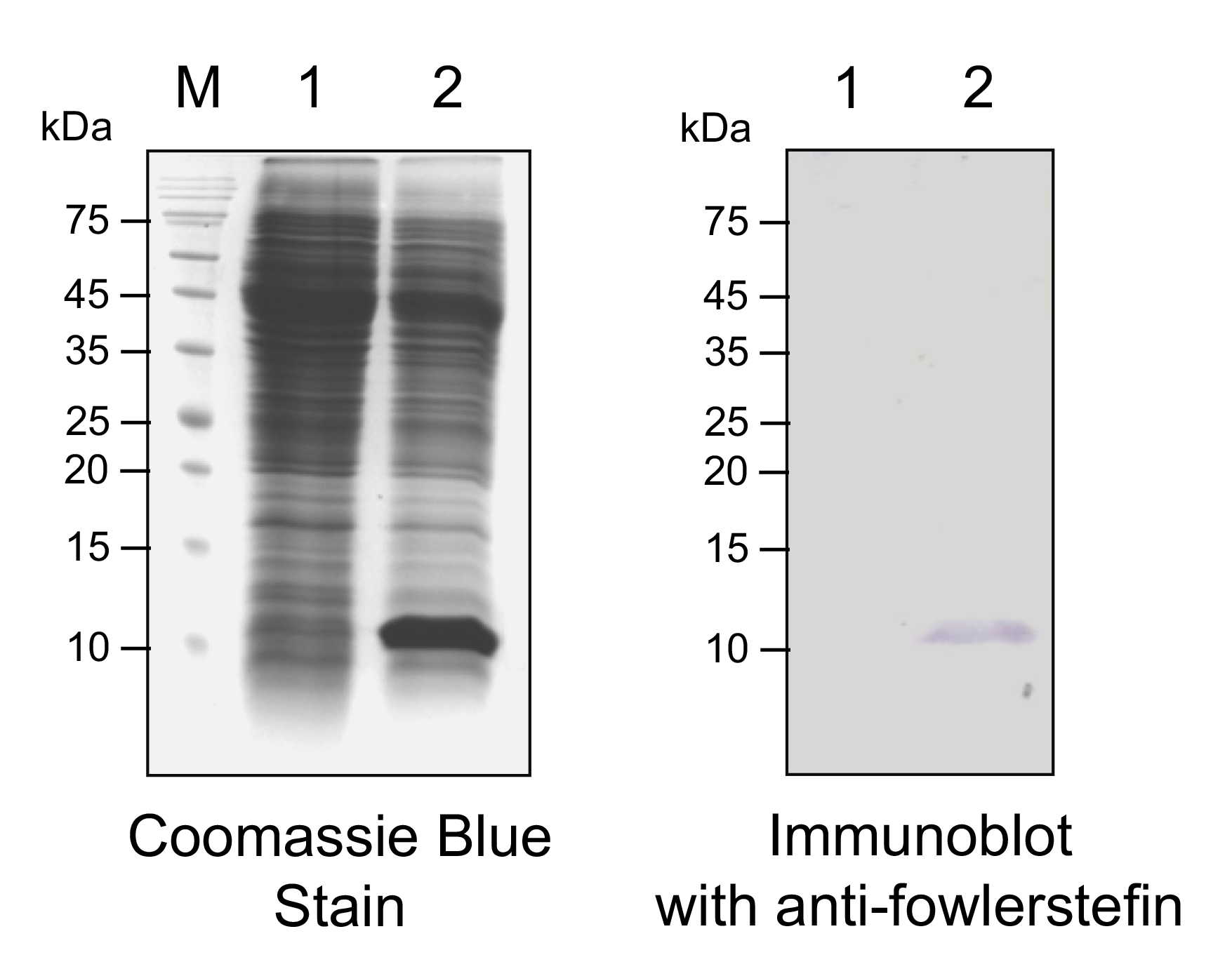

Supplement: Supplementary file 1 — Additional file 1: Figure S1. Sepficity of the anti-fowlerstefin. The anti-fowlerstefin was produced in mice by pertioneal immunizations. The specificty of the anitbody was confimed against E. coli lysate by immunblot analysis. Lane M: protein size marker; Lane 1: non-induced E. coli lysate (20 µg); Lane 2: IPTG-induced E. coli lysate (20 µg). [file 13071_2020_3909_MOESM1_ESM.tif]

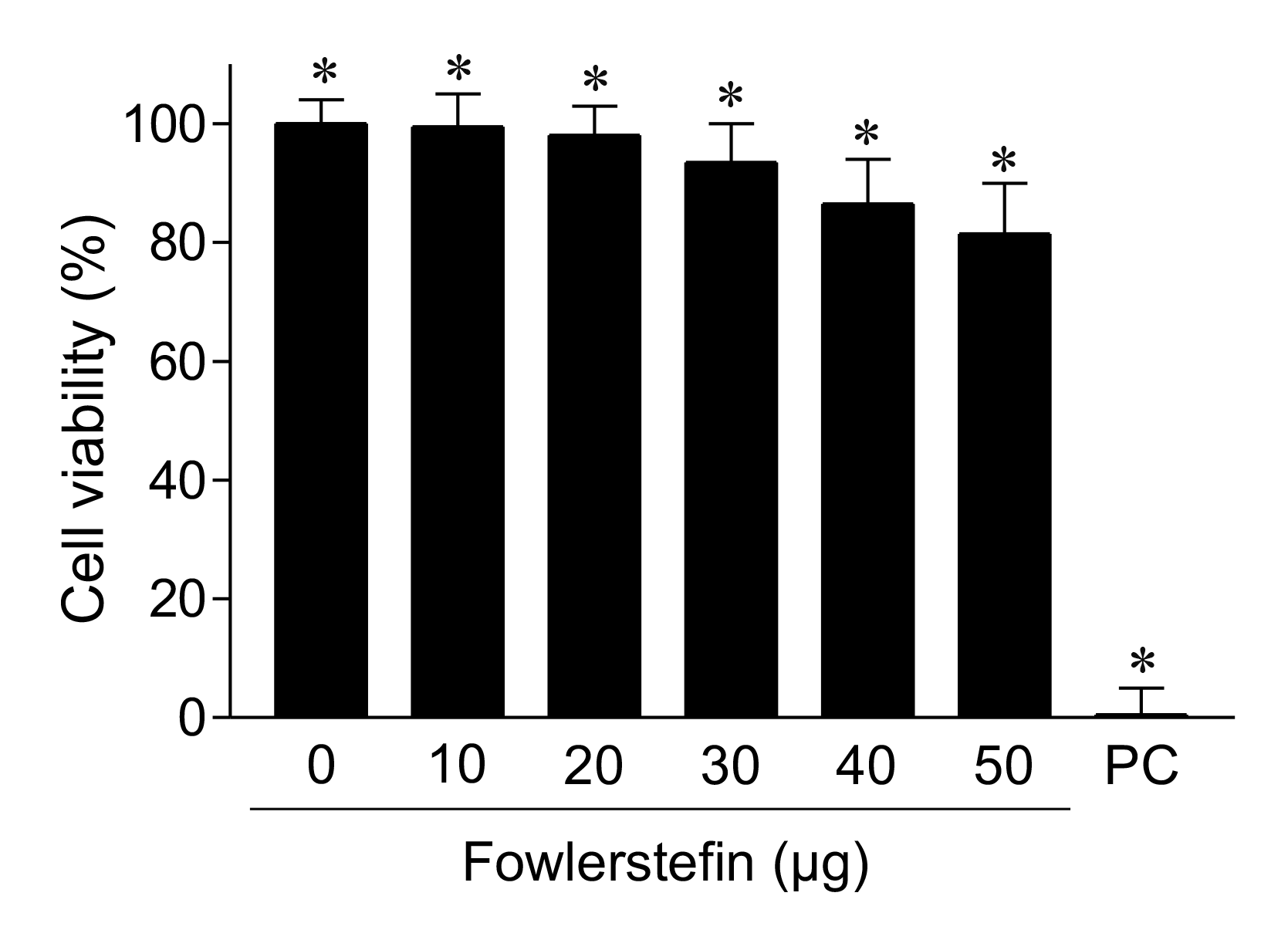

Supplement: Supplementary file 2 — Additional file 2: Figure S2. Cytotoxicity assay. BV-2 cells were treated with different concentrations of fowlersetfin and the cell cytotoxicity was assessed using the CytoTox 96® Non-radioactive cytotoxicity assay kit. PC: positive control represented 100% cell death. Assays were performed in triplicate and the mean and SD values were calculated. *P < 0.05. [file 13071_2020_3909_MOESM2_ESM.tif]
