# Supplementary material for: Fowlerstefin, a cysteine protease inhibitor of Naegleria fowleri, induces inflammatory responses in BV-2 microglial cells in vitro
Source: Parasit Vectors. 2020 Jan 29;13:41. doi: 10.1186/s13071-020-3909-6 (PMC6988287; doi:10.1186/s13071-020-3909-6)
Supplement: Supplementary file 3 — Additional file 3: Figure S3. Significant results from statistical analyses in this study. [file 13071_2020_3909_MOESM3_ESM.pdf]

Additional file 3: Text S1. Significant results from statistical analyses in this study.

Fig. 6b:
Ratio (TNF/GAPDH)
One-way ANOVA: $F_{(5, 12)} = 152$, $P < 0.0001$
Dunnett's multiple comparisons test:
NC vs. LPS: Mean diff = –0.763 (95% CI: –0.920 to –0.607), $P < 0.0001$
NC vs. fowlerstefin (3h): Mean diff = –1.10 (95% CI: –1.26 to –0.947), $P < 0.0001$
NC vs. fowlerstefin (6h): Mean diff = –1.11 (95% CI: –1.27 to –0.957), $P < 0.0001$
NC vs. fowlerstefin (9h): Mean diff = –1.11 (95% CI: –1.27 to –0.954), $P < 0.0001$
NC vs. fowlerstefin (12h): Mean diff = –1.27 (95% CI: –1.43 to –1.12), $P < 0.0001$
Ratio (IL-1α/GAPDH)
One-way ANOVA: $F_{(5, 12)} = 234$, $P < 0.0001$
Dunnett's multiple comparisons test:
NC vs. LPS: Mean diff = –0.897 (95% CI: –1.02 to –0.772), $P < 0.0001$
NC vs. fowlerstefin (3h): Mean diff = –1.05 (95% CI: –1.18 to –0.927), $P < 0.0001$
NC vs. fowlerstefin (6h): Mean diff = –1.07 (95% CI: –1.19 to –0.946), $P < 0.0001$
NC vs. fowlerstefin (9h): Mean diff = –1.16 (95% CI: –1.28 to –1.03), $P < 0.0001$
NC vs. fowlerstefin (12h): Mean diff = –1.28 (95% CI: –1.40 to –1.16), $P < 0.0001$
Ratio (IL-1β/GAPDH)
One-way ANOVA: $F_{(5, 12)} = 30.9$, $P < 0.0001$
Dunnett's multiple comparisons test:
NC vs. LPS: Mean diff = –0.648 (95% CI: –0.908 to –0.387), $P < 0.0001$
NC vs. fowlerstefin (3h): Mean diff = –0.856 (95% CI: –1.12 to –0.595), $P < 0.0001$
NC vs. fowlerstefin (6h): Mean diff = –0.798 (95% CI: –1.06 to –0.538), $P < 0.0001$
NC vs. fowlerstefin (9h): Mean diff = –0.888 (95% CI: –1.15 to –0.627), $P < 0.0001$
NC vs. fowlerstefin (12h): Mean diff = –0.949 (95% CI: –1.21 to –0.688), $P < 0.0001$
Ratio (IL-6/GAPDH)
One-way ANOVA: $F_{(5, 12)} = 155$, $P < 0.0001$
Dunnett's multiple comparisons test:
NC vs. LPS: Mean diff = –0.761 (95% CI: –0.912 to –0.610), $P < 0.0001$
NC vs. fowlerstefin (3h): Mean diff = –0.986 (95% CI: –1.14 to –0.835), $P < 0.0001$
NC vs. fowlerstefin (6h): Mean diff = –0.939 (95% CI: –1.09 to –0.788), $P < 0.0001$
NC vs. fowlerstefin (9h): Mean diff = –1.17 (95% CI: –1.32 to –1.02), $P < 0.0001$
NC vs. fowlerstefin (12h): Mean diff = –1.29 (95% CI: –1.44 to –1.14), $P < 0.0001$

Fig. 6c:
TNF (pg/ml)
One-way ANOVA: $F_{(5, 12)} = 209$, $P < 0.0001$
Dunnett's multiple comparisons test:
NC vs. LPS: Mean diff = –576 (95% CI: –635 to –516), $P < 0.0001$
NC vs. fowlerstefin (3h): Mean diff = –424 (95% CI: –483 to –364), $P < 0.0001$
NC vs. fowlerstefin (6h): Mean diff = –454 (95% CI: –513 to –394), $P < 0.0001$
NC vs. fowlerstefin (9h): Mean diff = –560 (95% CI: –620 to –501), $P < 0.0001$

NC vs. fowlerstefin (12h): Mean diff = –387 (95% CI: –446 to –327), $P < 0.0001$

IL-6 (pg/ml)

One-way ANOVA: $F_{(5, 12)} = 178$, $P < 0.0001$

Dunnett's multiple comparisons test:

NC vs. LPS: Mean diff = –131 (95% CI: –154 to –108), $P < 0.0001$

NC vs. fowlerstefin (3h): Mean diff = –73.9 (95% CI: –97.0 to –50.8), $P < 0.0001$

NC vs. fowlerstefin (6h): Mean diff = –150 (95% CI: –173 to –127), $P < 0.0001$

NC vs. fowlerstefin (9h): Mean diff = –205 (95% CI: –228 to –182), $P < 0.0001$

NC vs. fowlerstefin (12h): Mean diff = –180 (95% CI: –203 to –157), $P < 0.0001$

Fig. 7a:

Ratio (TNF/GAPDH)

One-way ANOVA: $F_{(8, 18)} = 75.8$, $P < 0.0001$

Dunnett's multiple comparisons test:

fowlerstefin vs. NC: Mean diff = 1.11 (95% CI: 0.928 to 1.30), $P < 0.0001$

fowlerstefin vs. LPS: Mean diff = –38.1 (95% CI: –59.0 to –17.1), $P = 0.0255$

fowlerstefin vs. SP600125 (1 μM): Mean diff = 144 (95% CI: 123 to 165), $P < 0.0001$

fowlerstefin vs. SP600125 (10 M): Mean diff = 246 (95% CI: 225 to 267), $P < 0.0001$

fowlerstefin vs. SB203580 (1 μM): Mean diff = 46.0 (95% CI: 25.1 to 66.9), $P = 0324$

fowlerstefin vs. SB203580 (10 μM): Mean diff = 283 (95% CI: 262 to 304), $P < 0.0001$

fowlerstefin vs. U0126 (1 μM): Mean diff = 61.9 (95% CI: 41.0 to 82.9), $P = 0.0004$

fowlerstefin vs. U0126 (10 μM): Mean diff = 275 (95% CI: 254 to 296), $P < 0.0001$

Ratio (IL-6/GAPDH)

One-way ANOVA: $F_{(8, 18)} = 335$, $P < 0.0001$

Dunnett's multiple comparisons test:

fowlerstefin vs. NC: Mean diff = 0.925 (95% CI: 0.829 to 1.02), $P < 0.0001$

fowlerstefin vs. LPS: Mean diff = –10.2 (95% CI: –18.2 to –2.09), $P = 0.9977$

fowlerstefin vs. SP600125 (1 μM): Mean diff = 122 (95% CI: 114 to 130), $P < 0.0001$

fowlerstefin vs. SP600125 (10 M): Mean diff = 187 (95% CI: 179 to 195), $P < 0.0001$

fowlerstefin vs. SB203580 (1 μM): Mean diff = 127 (95% CI: 119 to 135), $P = 0.0029$

fowlerstefin vs. SB203580 (10 μM): Mean diff = 190 (95% CI: 182 to 198), $P < 0.0001$

fowlerstefin vs. U0126 (1 μM): Mean diff = 118 (95% CI: 110 to 126), $P = 0.0005$

fowlerstefin vs. U0126 (10 μM): Mean diff = 163 (95% CI: 154 to 171), $P < 0.0001$

Fig. 7b:

TNF (pg/ml)

Dunnett's multiple comparisons test:

One-way ANOVA: $F_{(8, 18)} = 790$, $P < 0.0001$

fowlerstefin vs. NC: Mean diff = 312 (95% CI: 292 to 332), $P < 0.0001$

fowlerstefin vs. LPS: Mean diff = –38.1 (95% CI: –59.0 to –17.1), $P = 0.0004$

fowlerstefin vs. SP600125 (1 μM): Mean diff = 144 (95% CI: 123 to 165), $P < 0.0001$

fowlerstefin vs. SP600125 (10 M): Mean diff = 246 (95% CI: 225 to 267), $P < 0.0001$

fowlerstefin vs. SB203580 (1 μM): Mean diff = 46.0 (95% CI: 25.1 to 66.9), $P < 0.0001$

fowlerstefin vs. SB203580 (10 μM): Mean diff = 283 (95% CI: 262 to 304), $P < 0.0001$

fowlerstefin vs. U0126 (1 μM): Mean diff = 61.9 (95% CI: 41.0 to 82.9), $P < 0.0001$
fowlerstefin vs. U0126 (10 μM): Mean diff = 275 (95% CI: 254 to 296), $P < 0.0001$

IL-6 (pg/ml)

One-way ANOVA: $F_{(8, 18)} = 1826$, $P < 0.0001$

Dunnett's multiple comparisons test:

fowlerstefin vs. NC: Mean diff = 204 (95% CI: 196 to 212), $P < 0.0001$
fowlerstefin vs. LPS: Mean diff = –10.2 (95% CI: –18.2 to –2.09), $P = 0.0111$
fowlerstefin vs. SP600125 (1 μM): Mean diff = 122 (95% CI: 114 to 130), $P < 0.0001$
fowlerstefin vs. SP600125 (10 M): Mean diff = 187 (95% CI: 179 to 195), $P < 0.0001$
fowlerstefin vs. SB203580 (1 μM): Mean diff = 127 (95% CI: 119 to 135), $P < 0.0001$
fowlerstefin vs. SB203580 (10 μM): Mean diff = 190 (95% CI: 182 to 198), $P < 0.0001$
fowlerstefin vs. U0126 (1 μM): Mean diff = 118 (95% CI: 110 to 126), $P < 0.0001$
fowlerstefin vs. U0126 (10 μM): Mean diff = 163 (95% CI: 154 to 171), $P < 0.0001$

Fig. 8a:

Ratio (TNF/GAPDH)

One-way ANOVA: $F_{(6, 14)} = 40.5$, $P < 0.0001$

Dunnett's multiple comparisons test:

fowlerstefin vs. NC: Mean diff = 0.857 (95% CI: 0.616 to 1.10), $P < 0.0001$
fowlerstefin vs. LPS: Mean diff = –0.267 (95% CI: –0.518 to –0.0157), $P = 0.0363$
fowlerstefin vs. MG132 (1 μM): Mean diff = 0.190 (95% CI: –0.0610 to 0.441), $P = 0.1654$
fowlerstefin vs. MG132 (10 M): Mean diff = 0.557 (95% CI: 0.306 to 0.808), $P = 0.0002$
fowlerstefin vs. SR11302 (1 μM): Mean diff = 0.0700 (95% CI: –0.181 to 0.321), $P = 0.0324$
fowlerstefin vs. SR11302 (10 μM): Mean diff = 0.273 (95% CI: 0.022 to 0.524), $P = 0.0317$

Ratio (IL-6/GAPDH)

One-way ANOVA: $F_{(6, 14)} = 143$, $P < 0.0001$

Dunnett's multiple comparisons test:

fowlerstefin vs. NC: Mean diff = 1.14 (95% CI: 0.969 to 1.30), $P < 0.0001$
fowlerstefin vs. LPS: Mean diff = –0.127 (95% CI: –0.307 to 0.0534), $P = 0.2117$
fowlerstefin vs. MG132 (1 μM): Mean diff = 0.790 (95% CI: 0.610 to 0.970), $P < 0.0001$
fowlerstefin vs. MG132 (10 M): Mean diff = 0.960 (95% CI: 0.780 to 1.14), $P < 0.0001$
fowlerstefin vs. SR11302 (1 μM): Mean diff = 0.407 (95% CI: 0.227 to 0.587), $P = 0.0001$
fowlerstefin vs. SR11302 (10 μM): Mean diff = 0.837 (95% CI: 0.657 to 1.02), $P < 0.0001$

Fig. 8b:

TNF (pg/ml)

One-way ANOVA: $F_{(6, 14)} = 3049$, $P < 0.0001$

Dunnett's multiple comparisons test:

fowlerstefin vs. NC: Mean diff = 307 (95% CI: 296 to 319), $P < 0.0001$
fowlerstefin vs. LPS: Mean diff = –64.3 (95% CI: –76.9 to –51.7), $P < 0.0001$
fowlerstefin vs. MG132 (1 μM): Mean diff = 292 (95% CI: 279 to 305), $P < 0.0001$
fowlerstefin vs. MG132 (10 M): Mean diff = 315 (95% CI: 302 to 328), $P < 0.0001$
fowlerstefin vs. SR11302 (1 μM): Mean diff = 73.8 (95% CI: 61.2 to 86.4), $P < 0.0001$
fowlerstefin vs. SR11302 (10 μM): Mean diff = 210 (95% CI: 198 to 223), $P < 0.0001$

IL-6 (pg/ml)

One-way ANOVA: $F_{(6, 14)} = 338$, $P < 0.0001$

Dunnett's multiple comparisons test:

fowlerstefin vs. NC: Mean diff = 365 (95% CI: 326 to 404), $P < 0.0001$

fowlerstefin vs. LPS: Mean diff = –29.7 (95% CI: –72.1 to 12.8), $P = 0.2164$

fowlerstefin vs. MG132 (1 μM): Mean diff = 339 (95% CI: 296 to 381), $P < 0.0001$

fowlerstefin vs. MG132 (10 M): Mean diff = 367 (95% CI: 324 to 409), $P < 0.0001$

fowlerstefin vs. SR11302 (1 μM): Mean diff = 65.8 (95% CI: 23.4 to 108), $P = 0.0030$

fowlerstefin vs. SR11302 (10 μM): Mean diff = 239 (95% CI: 197 to 282), $P < 0.0001$
